# One‐Sidedness and the Inferior Function in *Coriolanus* and *Timon of Athens*

**DOI:** 10.1111/1468-5922.70023

**Published:** 2026-02-05

**Authors:** Sofie Qwarnström

**Affiliations:** ^1^ Ferney Voltaire France

**Keywords:** Carl Jung, inferior function, interdisciplinary, Introverted Thinking, one‐sidedness, personality, *Psychological Types*, Shakespeare, Carl Jung, Types Psychologiques, Shakespeare, Pensée Introvertie, fonction inférieure, personnalité, unilatéralisme, interdisciplinarité, Carl Jung, *Psychologische Typen*, Shakespeare, Introvertiertes Denken, Inferiore Funktion, Persönlichkeit, Einseitigkeit, Interdisziplinarität, Carl Jung, *Tipi Psicologici*, Shakespeare, pensiero introverso, funzione inferiore, personalità, unilateralità, interdisciplinarietà, Карл Юнг, психологические типы, Шекспир, интровертное мышление, подчиненная функция, личность, односторонность, междисциплинарность, Carl Jung, *Tipos Psicológicos*, Shakespeare, Pensamiento Introvertido, función inferior, personalidad, unilateralidad, interdisciplinario, 卡尔, 荣格, 心理类型, 莎士比亚, 内倾思维, 劣势功能, 人格, 片面性, 跨学科

## Abstract

For both Jung and Shakespeare, one‐sidedness is the fundamental tragic trait. Jung proposed that as an individual develops, they inevitably associate their identity with certain modes of perception and interaction, and that this leads to psychological polarization. The preferred function takes on a dominant role as ontological filter of the individual’s worldview, while the disregarded function remains unhewn and volcanic, left largely outside of conscious control. Jung insists that one‐sidedness, the over‐development of one side of the personality at the expense of the opposite “inferior” function, is a dangerous weakness. Likewise, Bradley (1905, p. 21) observes that in almost all of Shakespeare’s tragic protagonists, “we observe a marked one‐sidedness, … a fatal tendency to identify the whole being with one interest, object, passion, or habit of mind.” This article will outline the clear parallel between the structure of Shakespearean tragedy and the psyche as Jung understood it. It will do so through the juxtaposition of *Coriolanus* and *Timon of Athens,* in which the same functions are attributed opposite value. The contrast of these two plays seen through the lens of Jung’s *Psychological Types* will help us to understand the perils presented by one‐sidedness and the under‐theorized inferior function, and will flesh out the concepts of Introverted Thinking and Extraverted Feeling via amplification.

## Abstract (fr)

Pour Jung comme pour Shakespeare, l’unilatéralisme constitue le ressort tragique fondamental. Selon Jung, le développement de l’individu s’accompagne inévitablement d’une identification à certains modes de perception et d’action, entraînant une polarisation psychique. La fonction préférée s’impose comme filtre ontologique de la vision du monde, tandis que la fonction négligée demeure brute, volcanique, et échappe largement au contrôle conscient. Jung souligne que cet excès de développement d’un pôle de la personnalité, au détriment de la « fonction inférieure », représente une faiblesse périlleuse. De son côté, Bradley (1905, p. 21) remarque que, chez la plupart des héros tragiques de Shakespeare, « nous observons un unilatéralisme marqué, […] une tendance fatale à identifier tout l’être à un seul intérêt, objet, passion ou habitus mental ».

Cet article met en évidence le parallèle entre la structure de la tragédie shakespearienne et la dynamique de la psyché telle que la conçoit Jung. Il le fera en confrontant *Coriolan* et *Timon d’Athènes*, où les mêmes fonctions se voient attribuer des valeurs inverses. Ce contraste, envisagé à travers le prisme des *Types psychologiques* de Jung, éclaire les périls de l’unilatéralisme et de la fonction inférieure, tout en approfondissant, par amplification, les notions de Pensée introvertie et de Sentiment extraverti.

## Abstract (de)

Für Jung wie auch Shakespeare ist Einseitigkeit der grundlegende tragische Charakterzug. Jung postulierte, daß sich die Identität eines Individuums im Laufe seiner Entwicklung unweigerlich mit bestimmten Wahrnehmungs‐ und Interaktionsweisen verbindet, was zu einer psychologischen Polarisierung führt. Die bevorzugte Funktion übernimmt eine dominante Rolle als ontologischer Filter der individuellen Weltanschauung, während die inferiore Funktion unbearbeitet und vulkanisch bleibt und sich weitgehend der bewußten Kontrolle entzieht. Jung betont, daß Einseitigkeit, also die Überentwicklung einer Seite der Persönlichkeit auf Kosten der entgegengesetzten, 'minderwertigen' Funktion, eine gefährliche Schwäche darstellt. Auch Bradley (1905, S. 21) stellt bei fast allen tragischen Protagonisten Shakespeares 'eine ausgeprägte Einseitigkeit fest, … eine fatale Tendenz, das ganze Wesen mit einem einzigen Interesse, Objekt, einer einzigen Leidenschaft oder Geisteshaltung zu identifizieren.' Dieser Artikel skizziert diese deutliche Parallele zwischen der Struktur der Shakespeareschen Tragödie und der Psyche, wie Jung sie verstand. Dies geschieht durch die Gegenüberstellung von *Coriolan* und *Timon von Athen,* in denen denselben Funktionen entgegengesetzte Bedeutungen zugeschrieben werden. Der Kontrast dieser beiden Stücke, betrachtet durch Jungs *Psychologische Typen,* wird uns helfen, die Gefahren der Einseitigkeit und der untertheoretisierten minderwertigen Funktion zu verstehen und verdeutlicht desweiteren die Konzepte des Introvertierten Denkens und des Extravertierten Fühlens durch Amplifikation.

## Abstract (it)

Sia per Jung che per Shakespeare, l’unilateralità è fondamentalmente un tratto tragico. Jung ha suggerito che, sviluppando, l'individuo inevitabilmente associa la propria identità con certe modalità di percezione e interazione, e questo porta ad una polarizzazione psicologica. La funzione preferita acquisisce un ruolo dominante come filtro ontologico per la visione del mondo del soggetto, mentre la funzione che è trascurata rimane rozza e vulcanica, e si colloca prevalentemente fuori dal controllo della coscienza. Jung ha insistito che l’unilateralità, l’accrescimento di un lato della personalità alle spese della unzione opposta, “inferiore”, rappresenti una debolezza pericolosa. Similmente, Bradley (1905, p. 21) osserva che in quasi tutti i tragici protagonisti di Shakespeare, “osserviamo una marcata unilateralità, … una tendenza fatale a identificare tutto l’essere con un solo interesse, una sola passione, una abitudine della mente”. Questo articolo sottolinea questo chiaro parallelismo tra la struttura delle tragedie di Shakespeare e la psiche come concepita da Jung. Vengono contrapposti le opere *Coriolano* e *Timone d’Atene,* in cui alle medesime funzioni sono attribuiti valori opposti. Il contrasto tra queste due opere viene visto attraverso le lenti dei *Tipi Psicologici* di Jung, e questo ci aiuta a individuare i pericoli che sono presenti nell'unilateralità e nella sottovalutazione della funzione inferiore, oltre a considerare i concetti di pensiero introverso e sentimento estroverso attraverso l'amplificazione.

## Abstract (ru)

Как Юнг, так и Шекспир считали односторонность главным источником страданий. Юнг полагал, что по мере своего развития человек неизбежно идентифицируется с определенными способами восприятия и взаимодействия, что приводит к психологической поляризации. Предпочитаемая функция начинает играть ведущую роль и становится онтологическим фильтром для мировоззрения индивида, в то время как подчиненная функция остается в значительной степени примитивной и взрывоопасной, не поддаваясь влиянию сознательного контроля. Юнг подчеркивал, что односторонность, то есть чрезмерное развитие одной стороны личности за счет противоположной, «подчиненной», является в значительной мере слабостью. Аналогичным образом Брэдли (1905, с. 21) отмечает, что почти у всех героев трагедий Шекспира «мы наблюдаем заметную односторонность… фатальную тенденцию отождествлять все существо с единственным интересом, объектом, страстью или образом мыслей». В данной статье мы сравниваем пьесы «Кориолан» и «Тимон Афинскийо», где одним и тем же функциям приписывается противоположный смысл, и на их примере демонстрируем выраженное сходство между структурой шекспировской трагедии и психикой в понимании Юнга. Сопоставление этих двух пьес через призму психологической типологии Юнга помогает нам понять риски, связанные с односторонностью и недостаточным осознаванием подчиненной функцией, и посредством амплификации дополняет представления об интровертном мышлении и экстравертном чувстве.

## Abstract (es)

Tanto para Jung como para Shakespeare, la unilateralidad es el rasgo trágico fundamental. Jung propuso que, a medida que un individuo se desarrolla, inevitablemente asocia su identidad con ciertos modos de percepción e interacción, lo cual conduce a una polarización psicológica. La función preferida asume un rol dominante como filtro ontológico de la visión del mundo del individuo, mientras que la función desatendida permanece sin pulir y volcánica, quedando en gran medida fuera del control consciente. Jung insiste en que la unilateralidad, el desarrollo excesivo de un aspecto de la personalidad a expensas de la función «inferior» opuesta, es una debilidad peligrosa. Del mismo modo, Bradley (1905, p. 21) observa que en casi todos los protagonistas trágicos de Shakespeare «observamos una marcada unilateralidad, […] una tendencia fatal a identificar todo el ser con un único interés, objeto, pasión o hábito mental». Este artículo esbozará este claro paralelismo entre la estructura de la tragedia shakespeariana y la psique tal y como la entendía Jung. Lo hará mediante la yuxtaposición de *Coriolano* y *Timón de Atenas*, en las que a las mismas funciones se les atribuyen valores opuestos. El contraste de estas dos obras, visto desde la perspectiva de los Tipos Psicológicos de Jung, nos ayudará a comprender los peligros que entrañan la unilateralidad y la poca teorización de la función inferior, y nos permitirá ampliar los conceptos de Pensamiento Introvertido y Sentimiento Extravertido.

## Abstract (zh)

《科利奥兰纳斯》与《雅典的泰门》中的片面性与劣势功能

对荣格和莎士比亚而言, 片面性都是根本的悲剧特质。荣格提出, 随着个体的发展, 自我的身份认同会不可避免地联系上某些感知模式和互动模式, 而这将导致心理上的极化。个体所倾向的功能会扮演起一个主导的角色, 作为个体世界观的本体论过滤器, 而被忽视的功能则保持原始且如火山般躁动, 很大程度上游离于意识控制之外。荣格坚持认为, 片面性, 即人格某一面的过度发展, 会以牺牲对立的"劣势"功能为代价, 这是一种危险的缺陷。同样地, 布拉德利(1905, p. 21)发现, 在莎士比亚笔下, 几乎所有悲剧主人公身上, "我们都能看到一种显著的片面性 … … 一种致命的倾向, 即把完整的存在仅仅认同于某一种单一的兴趣、目标、激情或思维习惯。"本文将勾勒莎士比亚悲剧结构与荣格所理解的心理结构之间的清晰平行关系。通过将《科利奥兰纳斯》与《雅典的泰门》并置分析来阐明这一观点——在这两部作品中, 相同的心理功能被赋予了截然相反的价值取向。通过荣格心理类型理论对比这两部戏剧, 将帮助我们理解片面性, 及被理论低估的劣势功能带来的危险, 并通过扩充的方法, 来充实内倾思维与外倾情感的概念。

## The Paradox of Crippling Strength: One‐Sidedness, Hubris and Pride

Epistemological pride—the disproportionate commitment to a single mode of seeing—is central to both Jung’s typological framework and Shakespeare’s tragedies. According to Jung, people often come up against this stumbling block during the development of personality. The paradoxical obstacle to balanced development is the human tendency to elevate a certain “heroic ideal” or “heroic” aspect of oneself above other, equally valid ideals. The seductive appeal of a certain ideal “compels us to sacrifice everything else” (Jung, 1971, para. 167) and hinders us from giving due attention to the inferior function: “You achieve balance … only if you nurture your opposite. But that is hateful to you in your innermost core, because it is not heroic” (Jung, 2009, p. 263).

This pattern can also be discerned in Shakespeare’s plays. Haupt (1973, p. 28), with Waith (1962), argues that Shakespeare’s tragic protagonists are brought low by a flaw that has something heroic or “Herculean” about it: “in Antony poor judgment is integrated with a kind of bountiful greatness of spirit, and in Coriolanus a rigid pride is part of a heroic greatness which condemns any compromise with the practical aspects of life.” Bradley stresses the fine line between weakness and greatness in Shakespeare’s plays. It is precisely the protagonists’ strengths, “everything that is admirable” (Bradley, 1905, p. 29) in them, which, taken to an extreme, becomes their defect:

The tragic conflict … is a conflict of the spirit …. The essentially tragic fact is the self‐division … isolated powers face each other, making incompatible demands. The family claims what the state refuses, love requires what honour forbids. The competing forces are both in themselves rightful … but the right of each is pushed into a wrong, because it ignores the right of the other, and demands that absolute sway which belongs to neither alone, but to the whole of which each is but a part. (Bradley, 1962, p. 369)

As Friar Lawrence in *Romeo and Juliet* states, “Virtue itself turns vice, being misapplied.” (Shakespeare, 1597/2001, II.iii.21) The “fundamental tragic trait,” Bradley (1905, p. 20) pronounces, is not a particular quality, but “one sidedness,” the lack of right measure. Chesterton (1908/1909, p. 50) invites us to consider, for instance, the many instances in which the scientists’ search for truth is pitiless, and the humanitarian’s mission of pity is untruthful.

The current folk‐understanding of hubris is of a kind of boisterous arrogance. It would seem, however, that the classical notion of hubris was closer to the concept of a “virtue gone mad”, a problem of structural aberrancy: “the notion of dangerous violence inherent in hubris was often seen as a result of abundant, excessive wealth or fullness that engenders a blind folly” (Levine, 1993, p. 54). Michelini observes that the term *hubris* in ancient texts is used about plants that must be pruned because they suffer from a “superabundance of nurture” (Michelini, 1978, p. 38). The plant, she writes, cogently illustrates the paradox intrinsic to hubris: robust health and flourishing, if ill‐directed, may become aberrant and self‐destructive. (Michelini, 1978). The over‐ripeness of hubris is not necessarily related, therefore, to the behaviour we tend to associate with arrogance. For instance, an ideological commitment to the belief that kindness can solve all problems can be hubristic, as in Timon’s case.

Hubris, warns the chorus in *Oedipus Rex*, “breeds the *turannos* [tyrant]” (Sophocles, circa. 429 B.C./2020, para. 873). Ferguson (1958, pp. 46–51) notes that Homer, Herodotus, Aeschylus, Thucydides and Plato considered hubris as the chief sin. Spengler (1972, p. 3) points out hubris, “the principal fountain of bad judgment and disaster”, was seen as destructive of the unity and balance “of the cardinal virtues—courage, temperance, justice, and wisdom … all essential to political stability and the good life”. Hubris was the Greek precursor to Latin “superbia”, later termed “pride” (Dyson, 2006, p. 10).

For St. Thomas Aquinas, *pride* is “the movement by which the will is borne towards ends beyond its real limits” (Gilson, 1956, p. 57). For St. Augustine, pride is the essential prerequisite for all sin and consists of man’s drive to regard himself “as if he were himself light” (Augustine, circa. 413–426 A.D./2000a, para. 13). He puts forward the paradox that men fell in wanting to be like the gods, “By craving to be more, man becomes less. … For that is true which is written, ‘Pride goeth before destruction, and before honour is humility’” (Augustine, 413–426/2000a, para. 13). The elevation of one’s “own light” as the final arbiter, he writes, is the source from which the other sins (*hamartia*) will flow (Augustine, 413–426/2000a, para. 13). The psychological equivalent of the issue St. Augustine raises here might be put in the following terms: “The stubborn adherence to one’s own ego‐stance in defiance of the rest of the world (internal and external) is the unseen psychological step which precedes other more visible evils.” 1 In this article, I shall investigate the equivalence between Jungian one‐sidedness and what in Shakespeare’s plays has been termed *hubris*.

## Psychological Types

Jung’s theory of personality identifies eight motivational and perceptual drives (“functions”) which “filter” internal and external experience and constitute different modes of relating to the world: Introverted Feeling, Introverted Thinking, Extraverted Feeling, Extraverted Thinking, Introverted Sensation, Introverted Intuition, Extraverted Sensation, Extraverted Intuition (Table 1).

**Table 1:** The Eight Personality Functions

	Rational Functions		Irrational Functions	
	Thinking	Feeling	Sensation	Intuition
Introversion	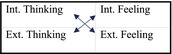		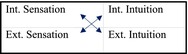	
Extraversion				

*Note*: The arrows represent “opposite” functions.

Jung writes that “The essential function of sensation is to establish that something exists, thinking tells us what it means, feeling what its value is, and intuition surmises whence it comes and whither it goes” (Jung, 1971, para. 553) 2. The introverted functions relate the process to an internal standard (Jung, 1971, para. 620–627), whereas the extraverted functions relate the process to the outside world (Jung, 1971, para. 563–67).

The intuition and sensation functions serve to register, integrate and organize information in one way or another (Jung, 1971, para. 953). Jung (1971, para. 953) refers to these two functions as the “irrational” functions (better understood as *arational or pre‐rational*), or “functions of perception” (Jung, 1971, para. 953). The “rational or judging” (Jung, 1971, para. 601) functions, thinking and feeling, serve to weigh this incoming information by reference to different standards of valuation. Together, the perceptual and judging functions constitute the eight‐function model of the psyche (Jung, 1971, para. 601).

**Figure 1:**
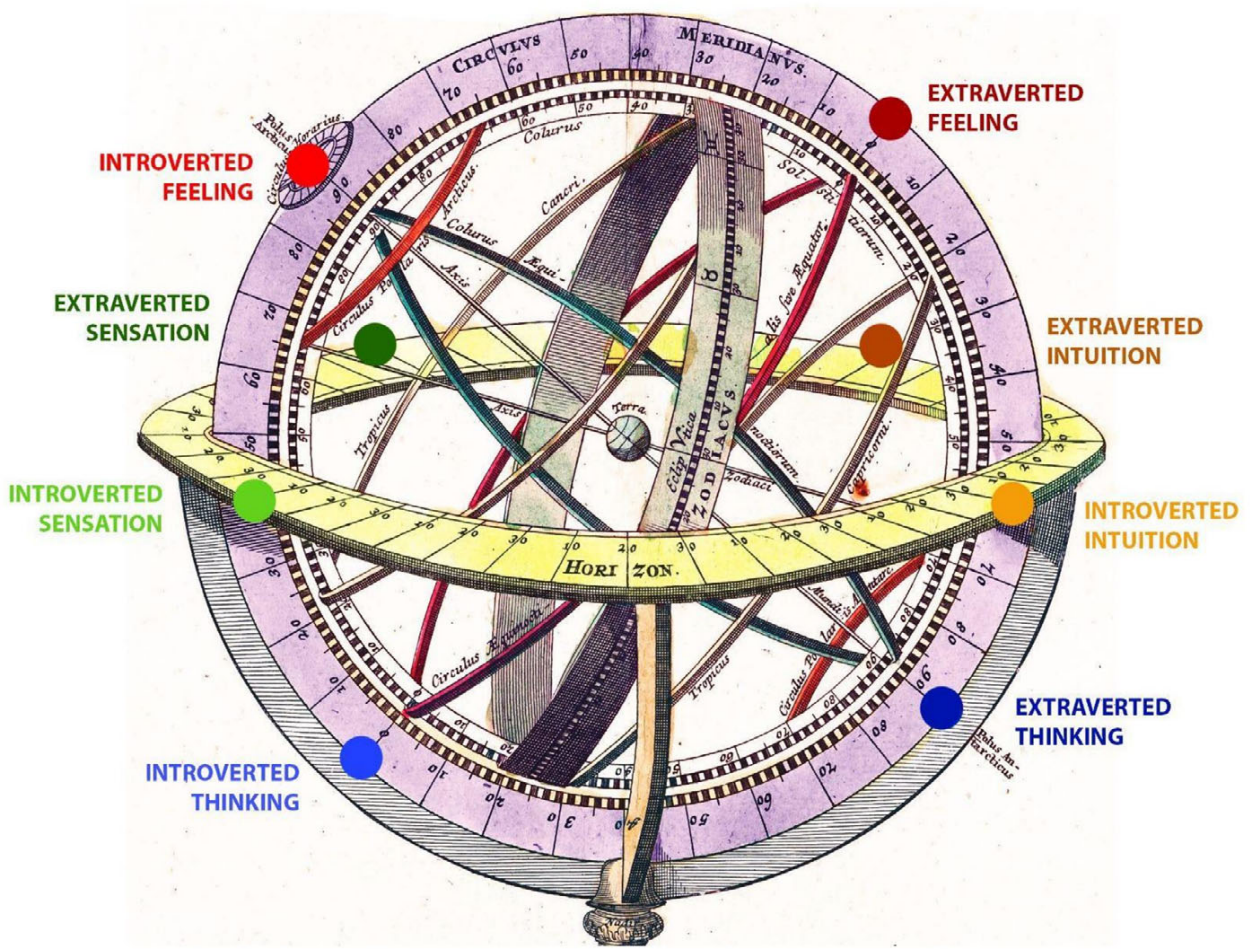
Jung’s eight functions represented as directions on an armillary sphere, where the meridian represents the spectrum of rational functions and the horizon represents the spectrum of irrational functions. Image adapted by author from Homann (1712) *Sphaerarum artificialium typica repraesentatio*.

Jung’s function‐types are not “boxes”—conclusive and static descriptions of whole personalities (Jung, 1971, p. 291)—but a terminology of points on a psychological compass; “just as arbitrary and just as indispensable” (Jung, 1971, para. 958). This compass allows us to refer to and describe the different cardinal directions of psychological specialization, and to understand the tensions between these extremes. It provides “a system of comparison and orientation” (Jung, 1971, para. 959). In an attempt to assist the conceptualization of the functions not as closed, static categories but as directions on a mobile and multi‐dimensional (Jung, 1971, para. 986) psychic compass, I have positioned each of the eight functions on an armillary sphere (Figure 1). I use the meridian and the horizon of this sphere to represent axes not in the sky but in the psyche. The meridian symbolizes the spectrum of rational functions and the horizon symbolizes the spectrum of irrational functions.

## One‐Sidedness

In Figure 1, “Earth” can be read as the ideal positioning of the ego at a balanced midpoint between the extremes, such that consciousness might reach evenly in each direction: if Feeling is required, the Feeling function is accessible, and likewise for the Thinking function. However, as Mahootian & Linné (2014, p. 399) say of their own model, my representation is limited: it is only “a static model of a dynamic system. In other words, these are only potentially conscious functions.”

An essential component of Jung’s theory is our tendency to one‐sidedness. According to him, it is well‐nigh impossible for a person to maintain such a balanced tension between the opposites as to have conscious access to all eight different modes of interpreting and reacting to the world: across individuals, Jung writes that “the basic psychological functions seldom or never all have the same strength or degree of development …. As a rule, one or the other function predominates” (Jung, 1971, para. 584). This one‐sidedness, he argues, is inevitable due to the finite nature of human attention. This finitude means that attention focused in one area will necessarily leave the opposite area in shadow. Because Thinking and Feeling, for example, are diametrically opposite ways of making decisions (Jung, 1971, para. 983), we are unable to attend to both at the same time: “Selection demands direction. But direction requires the exclusion of everything irrelevant. This is bound to make the conscious orientation one‐sided” (Jung, 1971, para. 694).

**Figure 2:**
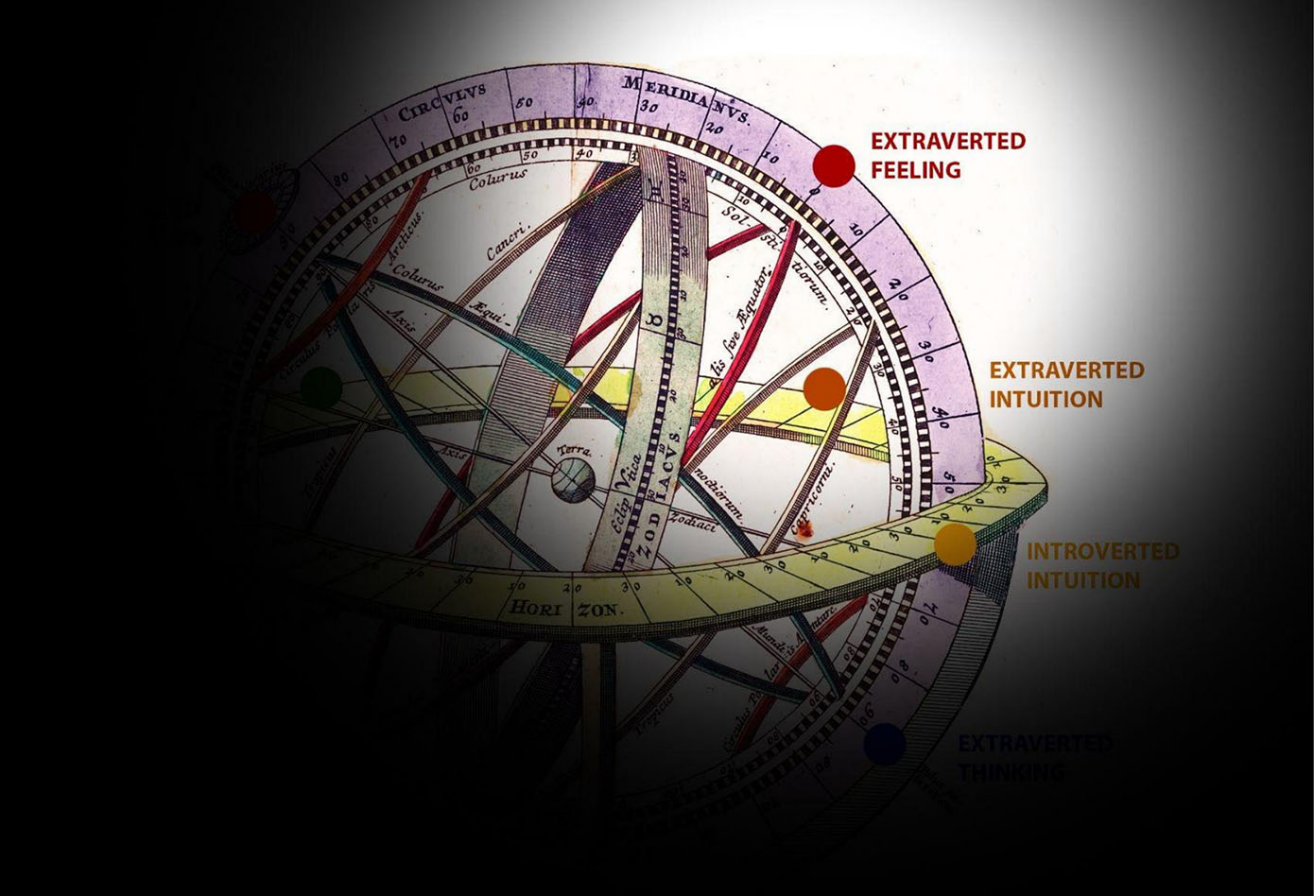
Armillary sphere of the functions in the psyche, partially illuminated by ego‐consciousness. Image adapted by author from Homann (1712) *Sphaerarum*.

Therefore, as a person develops, Jung theorizes that they tend to become “specialized” in relation to one or two functions (the “differentiated” or “superior” functions), which become a large part of the individual’s self‐image. 3 Jung theorizes that when one rationale is held up to the exclusion of another, it takes an authoritarian role in the psyche (Jung, 1971, para. 167) and becomes the decisive “governing principle” (Jung, 1971, para. 667) which orients consciousness. We may see here a parallel with the concept of hubris and its harmful “superabundance”. Figure 2 represents the functions of the psyche as experienced when the light of consciousness has a one‐sided Extraverted Feeling focus. The opposite function—Introverted Thinking in this case—is entirely wreathed in shadow (i.e., unconscious).

According to the specific nature of this imbalance, people can be broadly classified into “types” (Myers, 2019, p. 219). In Jung’s framework, a “Thinking type”, for instance, refers to a person with differentiated Thinking and inferior Feeling. This is not to say that such a person can only think and not feel. A Thinking type will feel just as much as a Feeling type, and their feelings may be genuine and incisive, but Feeling will generally be unconscious and the person will tend to have difficulty engaging with it in an adept and elegant way:

The superior function is always an expression of the conscious personality, of its aims, will, and general performance, whereas the less differentiated functions fall into the category of things that simply “happen” to one. These things need not be mere slips of the tongue … they can equally well be half or three‐quarters intended. (Jung, 1971, para. 482)

**Figure 3:**
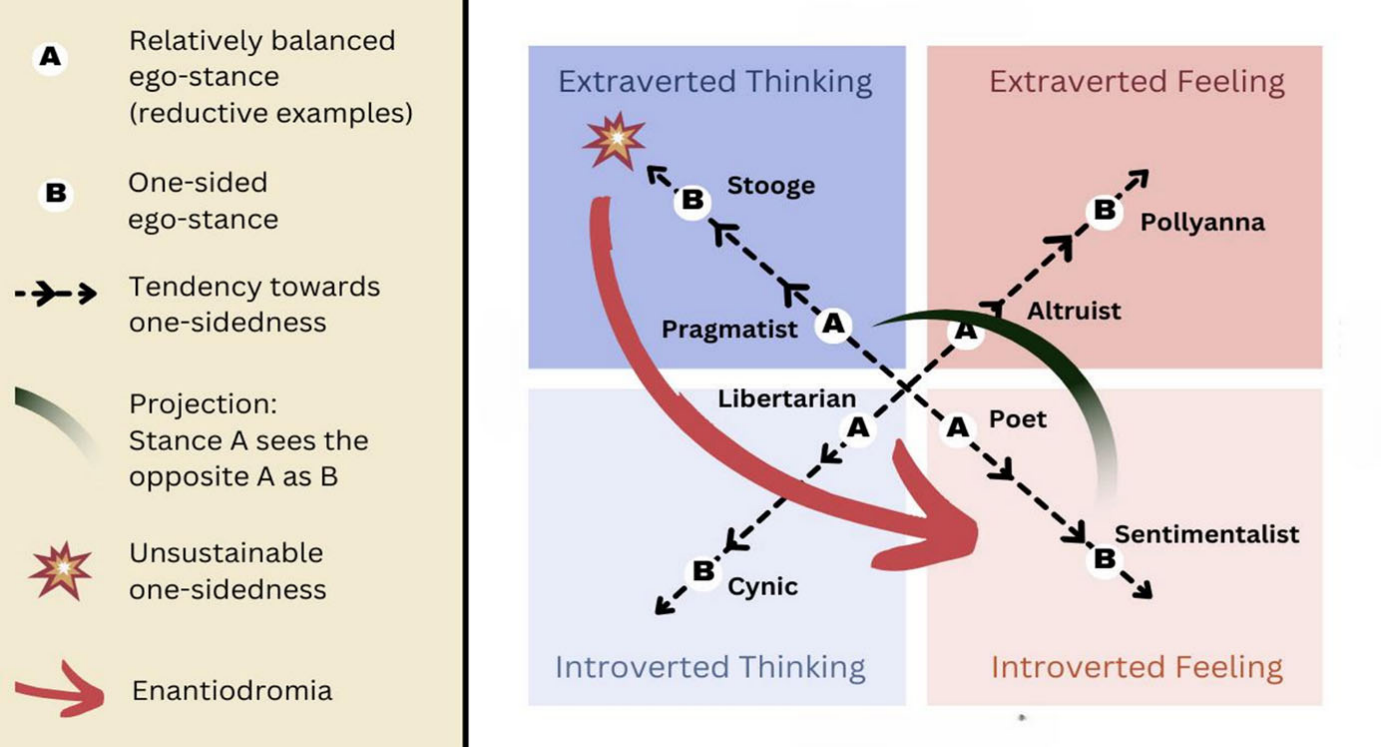
Example of enantiodromia away from a one‐sided conscious stance of Extraverted Thinking. Image by author.

In precise proportion to the over‐development of one function, the opposite (“inferior”) function sinks into the unconscious (Jung, 1971, paras. 105; 694). These rejected contents take on a compensatory attitude to consciousness and “form a counterweight to the conscious orientation” (Jung, 1971, para. 694). The more conscious one‐sidedness increases, the more this unconscious counter‐position grows in influence and strength. The result is noticeable psychical tension. When this tension becomes extreme, the personality “flips” over into an ego‐dystonic psychological extreme: that is, a mode of being markedly dissonant from the person’s habitual style of thought and behaviour. Jung calls this flip “enantiodromia”, after Heraclitus’ tenet that “everything eventually changes into its opposite” (Jung, 1971, para. 112). Jung describes this dynamic as a fundamental psychological law, a consequence of the self‐regulating tendency of opposites (Jung, 1967a, para. 111; 1969a, para. 425): in this new state, that which was formerly valued becomes worthless and that which was hitherto thought good comes to be seen as bad (Jung, 1971, para. 453). Figure 3 illustrates how one person’s demeaning projections onto an opposite personality (seeing opposite as “B” instead of “A”) reinforces both dislike, and the person’s own centrifugal temptation to become more one‐sided (B) oneself. Eventually, this one‐sidedness becomes unsustainable, results in breakdown, and enantiodromia takes place.

## Jung and Shakespeare

The archetypal nature of Shakespeare’s plays renders them a particularly interesting terrain on which to seek parallels with analytical psychology. Johnson (1765, p. viii) famously wrote that Shakespeare’s plays hold up a mirror to life, and that this verisimilitude accounts for their enduring popularity. For, he writes, “Nothing can please many, and please long, but just representations of general nature” (Johnson, 1765, p. viii). He specifies, however, that Shakespeare does not depict life as it is, but mirrors only that which is enduring and universal, and ignores much of that which is temporary and culturally specific (Johnson, 1765).

Like Johnson, Jung stressed that the tenacity of story patterns in our societal consciousness through time can be attributed to their usefulness and psychological veracity. The best narratives are passed across generations and speak “with a thousand voices” (Jung, 1966, para. 129) of that which we hold in common, the archetypal situations which transcend the individual. For instance, Jung argued that our oldest myths are constituted of primordial images which describe in condensed form the patterns of human life that span across the ages and are therefore a valuable source of insight into the human mind. Slochower (1970, p. 19) puts this well:

The ancient stories are retold, rewritten and transmitted as people find in them analogies to their own situation. … In this sense, myth is not something invented or fancied. It is rather a pictorial hypothesis about the nature of man … myths … enter the realm of reality in that they enable us to explain and predict events in the empirical world.

Jung (1953, para. 28) also contends that figurative language is the best and most succinct means of representing dynamics of the human psyche, and that “no intellectual formulation comes anywhere near the richness and expressiveness of mythical imagery.” Shakespeare’s plays, and the kaleidoscopic references within them, are rich tapestries of themes rooted in the mythology of classical antiquity (Bate, 1993; Burrow, 2013), in the folklore and ritual of Elizabethan England (Laroque*,* 1991; Wilson, 2009) and in Christian theology (Battenhouse, 1964; Gillies, 2013). The plays are richly entwined with the symbols and themes which are the meat and potatoes of mythology and folklore; mad kings, lost children, witches, wise fools, transformation, resurrection, riddles, ghosts, redemption, the crown as sacrificial burden and so on. Passed down, retold and rewritten through so many generations, the subject material of Shakespeare's plays is the rarefied quintessence of ancient narrative. From a Jungian perspective, therefore, Shakespeare’s plays are a particularly promising intermediary through which to explore the psychological wisdom we have inherited.

What’s more, Norris (2013, p. 206) notes that Shakespeare gives us a means of embodying Jung’s theoretical concepts through concrete images. On the stage, lived realities are brought down to earth— “to the messy contingencies of situated human conduct and choice.” (Norris, 2013, p. 206) This allows us to understand psychological theories in a direct and personal way, and to reflect on questions of ethics and relationship without getting lost in abstractions. As Jung (1969a, para. 468) writes, understanding is not an exclusively intellectual process, and the body of *Psychological Types* is an argument that understanding requires thinking, but also feeling, intuition and the input of the senses. I would like to suggest that the stage, appealing as it does to each of the functions, is uniquely suited to speak to these multiple dimensions of understanding.

In the remainder of this article, I shall flesh out Jung’s concept of one‐sidedness by contrasting two plays wherein the central protagonists display opposite forms of one‐sidedness. Coriolanus, whose inferior function is Extraverted Feeling, will be juxtaposed with Timon of Athens, whose inferior function is Introverted Thinking.

## *Coriolanus* and *Timon of Athens*

### Plot of *Coriolanus*

Coriolanus is a legendary Roman soldier, but a poor politician. Because of his opinionated advocacy of ruthless meritocracy and his lack of patience for the people’s complaints and inconstancy, Coriolanus is in disfavour with the plebeians. At his mother’s insistent prompting, Coriolanus seeks against his will to become consul but loses his temper in the process and is banished. He then turns against Rome, joins forces with Aufidius, his old Volsci enemy, and returns to sack his native city. At the last minute, his mother persuades him to seek a compromise in order to avoid the war and spare Rome. Aufidius then kills Coriolanus for turning against him.

### Plot of *Timon of Athens*

When Timon, a rich man of Athens, ignores Apemantus’ warnings and bankrupts himself through his extravagant generosity towards his fellows, servants and petitioners, he finds that his erstwhile friends are indifferent to his plight and content to watch him fall into ruin. The broken‐hearted Timon leaves Athens, curses the gods, mankind, and life itself, and encourages all that pass him by to destroy his city. He then commits suicide.

The principal problem of the protagonists of *Coriolanus* and *Timon of Athens* is an evaluative one. These plays are fundamentally about judgement and not perception. The issues in question relate to the proper evaluation of kindness, fairness, practicality, shrewdness and honesty. However, the way that the central characters represent value to themselves and make their decisions is one‐sided and therefore insufficient. When this state of imbalance becomes too flagrant, the repressed value system, as we shall see, begins to make an unconscious assault.

## Inferior Extraverted Feeling

As Bradley notes, the opening of Shakespearean tragedy functions as a prologue. It lays out the dominant patterns that have characterized the characters’ lives up until the play begins and the status quo that has presided in their world up until this point. The opening is designed to reveal the background of the hero’s world, which is already inhabited by the nemesis; “the force which is to prove fatal to the hero’s happiness” (Bradley, 1905, pp. 44–45). The witches in *Macbeth* introduce the insidious sphinx‐like desire inside of the protagonist to be king; the ghost in *Hamlet* embodies the Prince of Denmark’s intuition of the darkness within human nature and triggers the consequent struggle to find a reason to live, despite the “Cain” in mankind and himself. By first showing these gathering clouds, the effect is that “when we see the hero himself, the shadow of fate already rests upon him” (Bradley, 1905, p. 45): we are made aware from the start of the angle from which the hero’s undoing will come. The Greek word for this initial condition, “protasis”, implies in‐built consequences that will necessarily ensue: “the premise of a syllogism, the conditional clause,” from “proteinein”: that which stretches out before (Merriam‐Webster Editors, n.d.‐b). The protasis is the initial status quo, which has been gradually increasing in tension. The protasis depicts the kings’ initial hubristic one‐sidedness, hitherto harmless, but which begins to “heat the alembic”, to use an alchemical metaphor.

In both *Coriolanus* and *Timon*, this background theme is that of a hungry crowd, and the hero’s willingness, or not, to participate in community and to nourish the group. On one hand we have Timon, who throws lavish feasts for most of Athens. He gives so much that Apemantus says the mob of flatterers “eat” him (“Wilt dine with me, Apemantus?**/**No; I eat not lords” Shakespeare, [T.] 2001, I, i.244). In *Coriolanus,* on the other hand, the people starve and totter at the edge of rebellion, demanding corn, and naming Coriolanus as “chief enemy to the people”:

What authority surfeits on would relieve us: if they
would yield us but the superfluity …
we might guess they relieved us humanely;
but they think we are too dear: the leanness that
afflicts us, the object of our misery, is as an
inventory to particularise their abundance; our
sufferance is a gain to them. Let us revenge this with
our pikes, ere we become rakes: for the gods know I
speak this in hunger for bread, not in thirst for revenge.
(Shakespeare, [C.] 2001, I, i.14–22)

The plebeians say they do not ask for great charities from “authority” but only the scraps from their table, which they are denied. They accuse the rulers of withholding nourishment from them because this would lessen the rulers’ own store. A similar relationship holds between the superior and the inferior function in a state of one‐sidedness. Because we have no faith in our inferior function capacities, the superior function has a reflexive tendency to usurp the role of the inferior whenever possible. Von Franz (1971, p. 13) describes how the capacity for directed focus can become a limitation. When someone comes up against the inferior function “and experiences emotional shock or pain in confronting its real reactions … the superior function, like an eagle seizing a mouse, tries to get hold of the inferior function and bring it over into its own realm.” She illustrates this principle with the example of an introvert who continually substitutes relationship with others with internal rumination. This allows them to avoid taking the painfully flat‐footed steps into the foreign territory of their inferior function, but leaves them locked into an endless behavioural loop:

If an introvert, with his habitual way of introjecting, says he need not telephone Mrs so‐and‐so—she is just the symbol of his anima and therefore symbolic … he will never get to the bottom of his inferior function … By such a trick he simply tries to catch hold of his inferior function by means of his superior function … so as to maintain predominance … (von Franz, 1971, p. 7)

In this way, the “abundance” of the one function indeed results in the “leanness” of the other. The alternative would be for the superior function to sacrifice a little strength, for the personality to renounce some of its identity and to become, for a time, something of a “mixtum compositum”: “…a transitional stage where people are neither fish, nor flesh, nor good red herring!” (von Franz, 1971, p. 15)

Coriolanus expresses a fear of precisely this, the dilution of his identity through compromise. He therefore pushes for the maintenance of a state of affairs where the wisdom and political experience of the consuls rule, and disregards the wishes of the populace for what he sees as their own good:

What would you have, you curs,
That like nor peace nor war? … What’s the matter,
That in these several places of the city
You cry against the noble senate, who,
Under the gods, keep you in awe, which else
Would feed on one another?
(Shakespeare, [C.] 2001, I, i.164–190)

The people, of course, appreciate neither his opinion nor his abrasive approach.

Jung (1967a, para. 634) describes that the Introverted Thinking type will have no scruples engaging with controversial or hurtful ideas as long as the rationale is logically coherent:

… [He] will shrink from no danger in building up his world of ideas, and never shrinks from thinking a thought because it might prove to be dangerous, subversive, heretical, or wounding to other people’s feelings…. If in his eyes his product appears correct and true, then it must be so in practice, and others have got to bow to its truth.

Thus, in *Coriolanus*:

I’ll give my reasons,
More worthier than their voices. …
They ne’er did service for’t [corn]. Being pressed to th’ war…
They would not thread the gates. This kind of service
Did not deserve corn gratis. Being i’ th’ war,
Their mutinies and revolts, wherein they showed
Most valor, spoke not for them …
(Shakespeare, [C.] 2001, III, i. 1878–1886)

When engaged in their own area of expertise, Jung (1967a) writes that the readiness of the Introverted Thinking type to say everything they think necessarily provokes antagonism, which he does not have the interpersonal skills to respond to. More likely, the anger will draw his “primitive [inferior] affects … into acrimonious and fruitless polemics. Casual acquaintances think him inconsiderate and domineering. But the better one knows him, the more favourable one’s judgment becomes” (1967a, para. 635).

The one‐sidedness of Coriolanus’ stance is underlined by his categorical exclamation that the plebeians have nothing of worth at all to contribute to the political discussion:

[A democratic state‐of‐affairs] where gentry, title, wisdom
Cannot conclude, but by the yea and no
Of general ignorance … must omit
Real necessities, and give way the while
To unstable slightness: purpose so barr’d, it follows
Nothing is done to purpose. Therefore, beseech you, …
That love the fundamental part of state … at once pluck out
The multitudinous tongue; let them not lick
The sweet which is their poison.
(Shakespeare, [C.] 2001, III, i. 1905–1919)

Such a state, von Franz (1971, p. 20) describes, cannot last. She writes that if, when “the time comes for the development of the other functions,” one nevertheless continues to cling doggedly to old strengths, two things typically occur:

… the superior function degenerates like an old car that begins to run down and get worn out, and the ego becomes bored with it because everything you can do too well becomes boring; then, the inferior function, instead of appearing in its own field, tends to invade the main function, giving it an un‐adapted, neurotic twist.

Seen from this angle, the plebeians’ mutinous preparation for revenge parallels this brewing transition point and indicates something is stirring in Coriolanus’ known world. The threat of violence points to the danger of involuntary submersion under the forceful influence of the inferior function.

## The Hunger of the Masses: Communion and Eating

What does it mean that Timon and Coriolanus have opposing attitudes to “feeding the masses?” The masses are depicted in these two plays with imagery of the marketplace, the *agora,* the people, the plebeians, the revelling guests, and consistently accompanied by connotations of parade, fanfare, circus, revelry and Bacchus. In *Coriolanus*, there is emphasis on the undifferentiated nature of the masses; they are the “the many‐headed multitude,” (Shakespeare, [C.] 2001, II, iii.1439), “Hydra” (III, i.1847), “brats” (IV, vi.3130) and “children” (III, i.1763) to be “herded” (I, iv.1768).

Shakespeare draws a specific conceptual link between interpersonal relation and eating. The process of tuning into the felt values of the community is the characteristic of the Extraverted Feeling function, and indeed, the theme of social cohesion (harmony among the felt values of the community) appears in many Shakespeare plays in connection to eating and feasting. This link is both cultural and biological. Menenius, for instance, correlates Coriolanus’ pliancy to influence with the timing of his most recent meal 4:

He was not taken well; he had not dined:
The veins unfill’d, our blood is cold, and then
We pout upon the morning, are unapt
To give or to forgive; but when we have stuff’d
These pipes and these conveyances of our blood
With wine and feeding, we have suppler souls
Than in our priest‐like fasts …
(Shakespeare, [C.] 2001, V, i.3337–3343)

In his essay on *Coriolanus,* Wilson Knight (2002, p. 189) notes the same thing:

Menenius shares with Timon and Antony this strain of conviviality and warm‐hearted freedom of spirit in feasting. Which are just the qualities Coriolanus lacks: with him there is no surrendering of individuality to feasting or amusement or love. All is dominated by the one pride which knits his faculties to a steely centre of self‐consciousness sharp as a pin‐point; and as small and brittle.

Where Coriolanus sets himself up as a model and commands respect and gratitude, Timon’s more maternal supportive stance has the effect of fostering congruence and peace. Jung (1967b, p. 30) describes the opposites of singleness and communion in a way that closely parallels these roles:

Singleness is opposed to communion …
In communion let every man submit to others, that communion be maintained; for ye need it.
In singleness the one man shall be superior to the others, that every man may come to himself and avoid slavery …
Communion is depth.
Singleness is height.
Right measure in communion purifieth and preserveth.
Right measure in singleness purifieth and increaseth.
Communion giveth us warmth, singleness giveth us light.

To indulge, host, feed the nameless group, the “many‐headed multitude” is related to the loosening of boundaries; to love, self‐sacrifice, and a relaxation and sinking of the individual unity into the group. 5 The plebeians in *Coriolanus* are explicitly linked to the principle of interpersonal connection in their demand that Coriolanus respect the principle of harmony: “the price is to ask it kindly” (Shakespeare, [C.] 2001, II, iii.1507); “We shall be blest to [honour and advance Coriolanus], if he remember/A kinder value of the people than/He hath hereto prized them at.” (Shakespeare, [C.] 2001, II, ii.1293–1295)

In contrast, an orientation towards personal goals and values such as Coriolanus’ “solidifies” the individual unit and halts this dissolution. The plebeians perceive his individualism and defensive “precautionary measures” (Jung, 1967a, para. 634) as pride. Jung speaks of Introverted Thinking values “violating” the “object” by “abstracting” it: “All understanding as such, being an integration into general viewpoints, contains the devil’s element, and kills. It tears another life out from its own peculiar course and forces it into something foreign in which it cannot live” (Jung & Schmid‐Guisan, 2012, p. 141). Introverted Thinking concerns itself with meaning‐content, logos, divorced from form, from the mode of expression and realization of this content. Jung describes that one‐sided Logos attributes supreme value to abstract principles such as “State” and “Society”, while severing our connection to the felt‐experience (Jung, 1970a, para. 554). For instance, because of Coriolanus’ commitment to ideals, he strives to embody his abstract conception of “Rome”. But the Roman people themselves do not meet his standards of perfection, are not representatives of what his Rome should mean; they are not Romans “though calv’d i’ the porch o’ the Capitol” but barbarians “though in Rome litter’d” (Shakespeare, [C.] 2001, III, i, 2031–2033). Coriolanus cannot love what Rome truly consists of. 6

In asking “What is the city but the people?” (Shakespeare, [C.] 2001, II, i.1975), a consul, one of the people’s representatives, points out the paradox that you cannot wish for the betterment of something without first valuing the flawed thing already there. Jung’s statement that the introvert’s ideal “is a lonely island where nothing moves except what he permits to move” (1967a, para. 627) is echoed in the consul’s cry that Coriolanus’ fixation on his principles results in tyrannical expectations of others: “this viper/That would depopulate the city, and/Be every man himself” (Shakespeare, [C.] 2001, III, i.2069). The object will consequently “feel himself repulsed, and even belittled” (1967a, para. 633) by the introvert’s habit of disregarding them in favour of their own judgement, which, because of its unrelatedness, “appears cold, inflexible, arbitrary, and ruthless.” (1967a, para. 633)

In contrast, Jung (1967a, para. 557) describes that the extravert “has a positive relation to the object. He affirms its importance to such an extent that his subjective attitude is constantly related to and oriented by the object. The object can never have enough value for him.” Timon initially loves the people indiscriminately because they are Athenians. There is no conscious “condition” for his affection: “he is nowhere attached to anything, but soars above reality in a kind of intoxication; things are no longer seen as they are but are used merely as stimulants” (1967a, para. 475). When Apemantus is being contrarian, Timon tells him he will take no notice of Apemantus’ individual faults, but will welcome him despite who he is:

TIMON:
I take no heed of thee; thou’rt an Athenian,
therefore welcome: …
prithee, let my meat make thee silent.
APEMANTUS:
I scorn thy meat; ’twould choke me, for I should
ne’er flatter thee. O you gods, what a number of
men eat Timon, and he sees ’em not! It grieves me
to see so many dip their meat in one man’s blood …
(Shakespeare, [T.] 2001, I, ii.375–381)

The first warning in *Timon* of the danger underneath the surface appears in one of his first statements in the play. In a kind of vague, brief and unelaborated manner, he states a preference of portraits over men, “since dishonour traffics with man’s nature” and they only seem to be what they present themselves as. He likes the “pencill’d figures” better: they are exactly what they appear to be, “even such as they give out” (Shakespeare, [T.] 2001, I, i.197). This comment points us towards Timon’s subconscious awareness that his friends are deeply self‐interested. It also introduces the theme of an unbalanced preference for form over content, in contrast to the motif in *Coriolanus* of seeking content divorced from form.

## Pressure from the Inferior Function

It is characteristic of one‐sided Introverted Thinking to continually resist the pull to de‐centre from oneself and “dissolve” into the spirit of the group (Jung, 2009, p. 366). This resistance, this self‐enforced psychic solitude is a kind of self‐mortification which causes emotional starvation. (See Jung, 1970a, para. 275) I would like to propose that the despised and mutinous plebeians who are “resolved rather to die than to famish” (Shakespeare, [C.] 2001, I, i.5) can be seen as a representation of Coriolanus’ inferior function; his unconscious need to be in felt relation to his community.

The external representation of the inferior function in the angry plebians is both literary symbolism of his internal processes and a representation of literal external consequences of his “taciturn” and “acrimonious” (1967a, para. 635) demeanour, namely, the emotions he provokes in the community around him. This outer manifestation of his inner opposite relates to the algebra of cause and effect; in which the accumulation of all the things left undone or unsaid reaches a point where it becomes impossible to continue along the same comfortably familiar track: the unaddressed dimension of life, the ignored world of the inferior function, gains momentum and lashes back. Jung (1969b, para. 125) writes that he who does not take “the burden of completeness on himself” will find it “‘happening’ to him against his will in a negative form.” According to him, as we have seen, enantiodromia is a “psychological rule” (Jung, 1969b, para. 126). The neglected unconscious factor will eventually make itself felt with a force proportional to its former repression: “when an inner situation is not made conscious, it happens outside, as fate. That is to say, when the individual remains undivided and does not become conscious of his inner opposite, the world must perforce act out the conflict and be torn into opposing halves” (Jung, 1969b, para. 126).

The plebeians in *Coriolanus*, like the inferior function are “poor suitors” with “strong [i.e., pungent: worthless, unclean] breaths.” But the strong breath of the impotent will eventually result in expression via brute force: “They say poor/suitors have strong breaths: they shall know we/have strong arms too” (Shakespeare, [C.] 2001, I, i.51–53). An important distinction to make here is that I am not reading the plebeians as a representation of Extraverted Feeling, but Extraverted Feeling in an inferior state: they are seen through Coriolanus’ projections. Jung describes that:

*…* inferior extraversion detaches the individual entirely from his ego and dissolves him into archaic collective ties and identifications. He is then no longer “himself”, but sheer relatedness, identical with the object and therefore without a standpoint. The introvert instinctively feels the greatest resistance to this condition, which is no guarantee that he will not unconsciously fall into it. (1967a, para. 163)

Coriolanus’ vitriol stems from the fact that he is so identified with his ideas that any compromise of them seems to him to threaten his own disappearance. What’s more, on the unconscious level he is very afraid of the inferior Feeling part of himself because he has so little control over it.

While looking at Coriolanus and Timon of Athens side by side and reading the starving plebeians as an ignored personality dimension, one might be tempted to understand Timon’s feasting his guests on meat and wine as having a prosperous relationship with his unconscious. But on the contrary, Timon’s feasting of his friends—his developed Extraverted Feeling talent for interpersonal connection—is his conscious state of ease. It is not his guests who are representations of his unconscious, but the man who he ignores and bids be silent: Apemantus the cynic, an echo of Coriolanus, sits muttering in a corner and refuses to be fed. Like Coriolanus, 7 Apemantus is called a dog; an epithet he embraces for the dog’s qualities of being a guard, a spur, an enforcer of principles: “Away, unpeaceable dog, or I’ll spurn thee hence! /A: I will fly, like a dog, the heels o' the ass” (Shakespeare, [T.] 2001, I, i.316–317). Incidentally, the epithet of “cynic” originates from the “Greek *kynikos* ‘a follower of Antisthenes’, literally ‘dog‐like’, from *kyōn* ‘dog’” (Online Etymology Dictionary Editors, n.d.) The most famous Cynic was the philosopher Demosthenes of ancient Athens, who:

… hated students, emphasized self‐knowledge, discipline, and restraint, and held forth at a gymnasium named The Silver Hound in the old garden district outside the city. It was open to foreigners and the lower classes, and thus to Diogenes. Wits of the time made a joke of its name, calling its members stray dogs, hence cynic (dog‐like), a label that Diogenes made into literal fact, living with a pack of stray dogs, homeless except for a tub in which he slept. He was the Athenian Thoreau. (Davenport, 1995, pp. 16–17)

In the Merriam‐Webster Dictionary (n.d.‐a), the definition of *cynic* is listed as:

1. a fault‐finding captious critic especially: one who believes that human conduct is motivated wholly by self‐interest.
2. capitalized: an adherent of an ancient Greek school of philosophers who held the view that virtue is the only good and that its essence lies in self‐control and independence.

## The Introverted Thinking Fear of Feeling

Another parallel between the plays is that the fickle plebeians in *Coriolanus* and Timon’s traitorous guests are called “slaves”. Like Coriolanus, who says “… let me use my sword, I’d make a quarry/With thousands of these quartered slaves …” (Shakespeare, [C.] 2001, I, i.201–204), Timon’s steward Flavius, exclaims: “How many prodigal bits have slaves and peasants/This night englutted!” (Shakespeare, [T.] II, ii.1855–1856).

What the plebeians and Timon’s “suitors” have in common is that they do not follow values of their own. Coriolanus sees the group as an unpredictable and capricious force: “such as cannot rule nor ever will be ruled” (Shakespeare, [C.] 2001, I, i.1777–1778); a “beast with many heads” (Shakespeare, [C.] 2001, IV, i.1522–1523) which must be subdued by strict law:

He that trusts to you,
Where he should find you lions, finds you hares;
… You are no surer, no,
Than is the coal of fire upon the ice … Trust you?
With every minute you do change a mind
And call him noble that was now your hate,
Him vile that was your garland.
(Shakespeare, [C.] 2001, I, i.171–186)

Coriolanus’ accusation is not baseless; the plebeians begin a riot, join a battle, vote for Coriolanus and then push for his exile, and later rescind each of these initiatives. Coriolanus speaks in uncharacteristically emotional terms about his fear of them, saying it was a mistake to give their spokespeople positions of power and that his “soul aches to know” (Shakespeare, [C.] 2001, III, i.1863) whether, if the two equal sides are given equal representation and the principles of the state were laxened, the emotional chaos of the crowds would win out over the rule of principle. Jung (1970b, para. 21) offers us a clue as to the nature of Coriolanus’ fear: “One is usually afraid of things that seem to be overpowering. But is there anything in man that is stronger than himself?”:

If we submit such a case to an association experiment, we soon discover that he is not master in his own house. His reactions will be delayed, altered, suppressed, or replaced by autonomous intruders … very often unconscious even to himself … just as if the complex were an autonomous being capable of interfering with the intentions of the ego.

Jung (Jung & Schmid‐Guisan, 2012, p. 160) describes the Introverted Thinking principle, saying: “I want to purge my thinking of all that is erratic and unaccountable, of all pleasure and unpleasure caused by personal feeling, and raise it to the height of justness and the crystal‐clear purity of the universally valid idea.” The motivation for Coriolanus’ and Apemantus’ neglect of communal feeling is half due to the conscious will to remain true to their principles, and half due to unconscious fear of the feeling arena: “Because it is difficult to remain true to our principles amidst all the ardour of the feelings, we adopt the more comfortable expedient of making the character more secure by blunting them” (Schiller in Jung, 1967a, para. 635).

Indeed, despite all his lip service to willpower and his focus on personal principle, there is an indication in *Coriolanus* that this need to repress feeling stems in fact from a particular vulnerability to feeling. It is whispered of Coriolanus that he is inordinately influenced by his mother (Shakespeare, [C.] 2001, I, i.31–32). Indeed, she confirms this (“my praises made thee first a soldier” [Shakespeare, [C.] 2001, III, ii.2295]). He obeys all her demands throughout the play, whether he wishes to or not. Jung (1967a, para. 634) writes that the Introverted Thinking type’s conscious self‐directedness is in “strange contrast” to his “suggestibility to personal influences.” Due to unilateral focus on the pursuit of ideas, “his relation to people and things is secondary” (1967a, para. 634). The resulting “innocence” in the Extraverted Feeling realm means he “has only to be convinced of a person’s seeming innocuousness to lay himself open to the most undesirable elements. They seize hold of him from the unconscious. He lets himself be brutalized and exploited in the most ignominious way” (1967a, para. 634). This is what Coriolanus is guarding against.

Coriolanus therefore ties his identity to his will, as does Apemantus. Apemantus in *Timon* refuses to consume Timon’s meat and wine, accepting only water and gnawing a carrot he brought along with him. He, like Coriolanus, is led by principles of self‐control and independence, as if the object were striving to gain power over him. Coriolanus’ resistance to community, for instance, causes his soldiers, despite their love for him, to fear even to presume to show themselves as his friends. But, should he say the word, they follow him to battle as eagerly as “conies” (rabbits) emerging from their hovels after rain (Shakespeare, [C.] 2001, IV, v.2981). To both of these Introverted Thinking characters, to be weak‐willed is the worst insult there is (“I hate thee worse than a promise‐breaker” [Shakespeare, [C.] 2001, I, viii.737–738]).

By the logic of will as supreme value, we come to understand why Coriolanus and Apemantus call the weak‐willed masses “slaves”. To take a theological parallel, St. Augustine (circa. 413–426 A.D./2000b, para. 3) asserts that what makes true slavery is not obedience to an external master, but the absence of self‐control: “the good man, although he is a slave, is free; but the bad man, even if he reigns, is a slave … not of one man, but, what is far more grievous, of as many masters as he has vices.” Milton (1835, p. 917) too, warns that unchecked desires have the power to enslave:

Unless you will subjugate the propensity to avarice, to ambition, and sensuality … you will find that you have cherished a more stubborn and intractable despot at home, than you ever encountered in the field; and even your very bowels will be continually teeming with an intolerable progeny of tyrants.

The image of teeming bowels here connotes both pregnancy and parasites, connoting spiritual prostitution. The image implies that where there is no firm and reflective government over oneself and one’s loyalties, individuals allow themselves through their laxity to become servile to their own drives, and through them, the breeding ground for whatever opportunistic ruler has the know‐how to manipulate and infect their desires. It is not socio‐economic status that determines this kind of slavery and freedom. It is a question of choice and self‐determination; the lack of ability to dictate one’s own fate above the clamour of the instincts is what renders a person a true slave, or, as the masses are also termed in both plays, animals, children, etc. A recurrent theme in *Coriolanus* is the idea of self‐creation, of not relying on outer help:

I’ll never … Be such a gosling to obey instinct, but stand
As if a man were author of himself,
And knew no other kin.
(Shakespeare, [C.] 2001, V, iii.3528–3531)

Coriolanus calling the plebeians “slaves” is projection; it is what Extraverted Feeling means to *him*—for his ignored inferior function exerts such a strong pull on him that he senses it would not take much for it to overpower him. In compensation for this weakness, Coriolanus devotes himself to logos so entirely that he is repeatedly referred to as seeking or attaining godhead. 8 As Jung (1970a, para. 554) remarks though, pure logos is just as dangerous as pure eros.

The danger of one‐sidedness is just as present on both sides of the Extraverted Feeling/Introverted Thinking spectrum. Coriolanus utterly subjugates himself to the rule of his personal logical framework, but in his devotion to the tyrannical rule of the mind, he does not consult external perspectives and leaves no space for the values of others. Timon, on the other hand, abandons himself to his sympathy for others and is ruled by another kind of tyrant. Both characters conflate self‐interest with love, but where Coriolanus sees nothing but self‐interest and therefore banishes both from his consciousness, “starving” his world, a macrocosm of the state of his soul, in the process, Timon shuts the idea of self‐interested desires out of his awareness and therefore lets everyone in in the name of *philia*. In this way, he inadvertently gives himself to the masses to be “eaten”, as Coriolanus fears he would be if he were to cede even an inch to the plebeians. The “psychomachic” role Apemantus plays in the *Timon* universe is therefore the inverse of the plebeians’ role in *Coriolanus*; Apemantus is the spokesman of the split‐off unemotional and measuring part of Timon’s soul (Introverted Thinking), the part that observes without a feeling engagement with others. This part, like a watchdog, is a safety system that Timon insistently ignores, muffling it with his frenzied and unconditional trust in humankind. Jung (1967a, para. 973) describes this dynamic in the Extraverted Feeling type as follows: “He has no secrets he has not long since shared with others. Should something unmentionable nevertheless befall him, he prefers to forget it. Anything that might tarnish the parade of optimism and positivism is avoided.”

## Conclusion

If the hunger of the mob in these plays is read as emotional hunger, Timon is so ready to give of his energy and care to others that he is wide open and is left with nothing, and Coriolanus gives so little, is so efficiently barricaded, that he is like a dam, holding back immense pressure and causing drought. Coriolanus’ one‐sided approach is extremely supportive in a technical sense (e.g., he risks his personal well‐being to protect his country), but he is as deaf to the feelings of the people as he was to his own. Timon, at the opposite end of the spectrum, cannot separate himself from his connection to his fellows enough to be able to recognize their mercenary intent. The conflict of worldviews in these plays mirrors the tug of war between the Extraverted Feeling prioritization of interpersonal integration and the Introverted Thinking concern for maintaining impersonal objectivity in order to retain a strong capacity for individual self‐direction. At the end of both plays, it will be revealed that the two opposites cannot sustainably exist in isolation from the other (Qwarnström, in press). It is useless to have independent principles without considering communal harmony, just as it is worthless to consider communal harmony without the anchor of independent principles.

We have seen how sustained one‐sidedness causes the individual’s personality framework to become rigid and incapable of venturing outside a narrowly defined area of psychological “expertise”. Incapable of rupture and repair, the individual then becomes increasingly fragile and dependent on a certain lifestyle. This sets the stage for fanaticism and dissociation, where everything “opposite” to the rigidly held view is set up as a personal insult. We project our own repressed traits onto others, and our disassociation from this “foreign” stance causes us to see our hatred not as a consequence of our own weakness, but as a justified reaction to evil in others. Blind to the shortcomings of our own personality, we allow ourselves to fear, hate and dehumanize opposing views. On a personal scale, one‐sidedness fuels dangerous cycles of judgement, tunnel vision, rigidification, division and condemnation. On the national scale, zealous hubris quickly becomes catastrophic. Jung warned that our most urgent priority today must not be to perfect our technical capacity to control nature, but to cast a cold and searching gaze into the mirror in order to concern ourselves with the character of those who wield this deadly power.

## Footnotes

^1^
Hayek puts it this way: “Never will man penetrate deeper into error than when he is continuing on a road which has led him to great success” (von Hayek, 1941, p. 9).

^2^
For Jung’s full description of the functions, see Jung, 1971, paras. 577–619; 628–665.

^3^
Jung (1971, para. 560) also suggests that there may be a hereditary component to function‐preferences.

^4^
The literal truth of this connection is suggested by the findings of Danziger, Levav & Avnaim‐Pesso (2011) which indicate that “guilty” verdicts in court reduce significantly after lunch.

^5^
Jung specifies that eros strives towards connection, while Logos strives toward perfection: “Eros is an interweaving; Logos is differentiating knowledge, clarifying light. Eros is relatedness, Logos is discrimination and detachment” (Jung, 1968, para. 60); “Eros, the great binder and loosener … could be expressed in modern terms as psychic relatedness, and that of Logos as objective interest” (Jung, 1970a, para. 275).

^6^

1971, para. 630: introverted thinking “easily gets lost in the immense truth of the subjective factor, … apparently with an eye to real or at least possible facts, but always with a distinct tendency to slip over from the world of ideas into mere imagery.”

^7^
—of whom the citizens say, “he’s a very dog to the commonalty” (Shakespeare, [C.] 2001, I, i. 24).

^8^
E.g. “You speak o’ th’ people / As if you were a god to punish” (Shakespeare, [C.] 2001, III, i.1829–1830); “He is their god; he leads them like a thing / Made by some other deity than Nature” (IV, vi.3127–3128).
